# Neuroprotective effects of Wharton’s jelly-derived mesenchymal stem cells on motor deficits due to Parkinson’s disease 

**DOI:** 10.22038/IJBMS.2021.54091.12159

**Published:** 2021-09

**Authors:** Maryam Sadat Jalali, Alireza Sarkaki, Yaghoub Farbood, Seyed Saeed Azandeh, Esrafil Mansouri, Mohammad Ghasemi Dehcheshmeh, Ghasem Saki

**Affiliations:** 1 Persian Gulf Physiology Research Center, Ahvaz Jundishapur University of Medical Sciences, Ahvaz, Iran; 2 Department of Physiology, Faculty of Medicine, Ahvaz Jundishapur University of Medical Sciences, Ahvaz, Iran; 3 Department of Anatomical Sciences, Cellular and Molecular Research Center, Faculty of Medicine, Ahvaz Jundishapur University of Medical Sciences, Ahvaz, Iran; 4 Department of Immunology, Faculty of Medicine, Ahvaz Jundishapur University of Medical Sciences, Ahvaz, Iran

**Keywords:** Dopamine, L-dopa, Mesenchymal stem cells, Parkinson’s disease, Rat, Tyrosine hydroxylase

## Abstract

**Objective(s):**

Human Wharton’s jelly-derived mesenchymal stem cells (hWJ-MSCs) have been recognized as a potential tool to replace damaged cells by improving the survival of the dopaminergic cells in Parkinson’s disease (PD). In this study, we examined the effects of hWJ-MSCs and associated with L-dopa/carbidopa on motor disturbances in the PD model.

**Materials and Methods:**

PD was induced by injection of 6-hydroxydopamine (6-OHDA) (16 μg/2 μl into medial forebrain bundle (MFB)). Sham group received a vehicle instead of 6-OHDA. PD+C group received hWJ-MSCs twice on the 14^th^ and 28^th^ days post PD induction. PD+C+D group received hWJ-MSCs and also L-dopa/carbidopa (10/30 mg/kg). PD+D group received L-dopa/carbidopa alone. Four months later, motor activities (the parameters of locomotor and muscle stiffness) were evaluated, dopaminergic neurons were counted in substantia nigra pars compacta (SNc), the level of dopamine (DA), and tyrosine hydroxylase (TH) were measured in the striatum.

**Results:**

Data indicated that motor activities, the number of dopaminergic neurons, and levels of DA and TH activities were significantly reduced in PD rats as compared to the sham group (*P*<0.001). However, the same parameters were improved in the treated groups when compared with the PD group (*P*<0.001 and *P*<0.01, respectively).

**Conclusion:**

The chronic treatment of PD rats with hWJ-MSCs and L-dopa/carbidopa, improved motor activity, which may be the result of increased TH activity and due to released DA from dopaminergic neurons.

## Introduction

Parkinson’s disease (PD) is an age-dependent neurodegenerative disorder, which affects 1-2% of the world’s people that are over 60 years old. Dopamine (DA) depletion in the substantia nigra pars compacta (SNc) causes symptoms such as resting tremor, rigidity, bradykinesia, and postural instability (1-3). The enzyme, tyrosine hydroxylase (TH), catalyzes the formation of L-dopa, the rate-limiting step in the biosynthesis of DA, thereby directly linking PD with TH (4). Certainly, early loss of TH activity followed by a decline in TH protein is considered to contribute towards DA deficiency and phenotypic expression in PD (4, 5). The PD etiology is not still completely clear, but the creation of reactive oxygen species (ROS), which leads to oxidative stress and eventually neuronal death, could contribute to the disease pathology (6, 7). It has been suggested that treatment of Parkinson’s patients with stem cell transplantation (SCT) is more effective than drugs such as levodopa because these cells have the potential to differentiate and proliferate into different types of cells (1, 3, 8). In recent studies, the adipose-derived mesenchymal stem cells (MSCs) used in the PD model, could progress some movement syndromes (9-11). Moreover, it is established that injected human adipose-derived stem cells (hASC) into the tail vein of rats could pass through the blood-brain barrier and migrate into the damaged brain zones (12, 13). However, human Wharton’s jelly-derived mesenchymal stem cells (hWJ-MSCs) are more useful than other types of stem cells and can secrete cytokines to expand neural cells (14, 15). For a better understanding of the mechanisms of neurodegeneration and therapeutic approaches in PD patients, animal models of PD have been used in many studies (16, 17). Therefore, in order to obtain a PD treatment, the therapeutic applications of hWJ-MSCs and drug therapy, alone and in combination with each other, were evaluated by certain parameters such as motor disorders and dopaminergic neuronal count in SNc, and the levels of DA and TH in the striatum in a PD model.

## Materials and Methods

***Animals***

Fifty adult male Wistar rats (250-300 g) were taken from Ahvaz Jundishapur University of Medical Sciences (AJUMS) central animal Lab (Ahvaz, Iran). All experiments were approved by the Local Animal Ethics Committee of AJUMS (Code: IR.AJUMS.REC.1396.685). All rats were handled for 3 days (daily 5 min) before the tests then were divided randomly into the 5 groups (n=10) as follows:

1) Sham group, received a vehicle of 6-Hydroxydopamine (6-OHDA) (2 μl normal saline containing 0.01% ascorbic acid, SIGMA-Germany) into right medial forebrain bundle (MFB) through stereotaxic surgery.

2) PD group, received 6-OHDA (SIGMA-Germany) (16 μg/2 μl normal saline containing 0.01% ascorbic acid) into right MFB through stereotaxic surgery. 

 3) PD+C group, MFB-lesioned rats, received 1×10^6^ of hWJ-MSCs (injected twice on the 2^nd^ and 4^th^ week after PD induction through a tail vein) (1). 

4) PD+C+D group, MFB-lesioned rats, received a combination of hWJ-MSCs (1×10^6^ cells injected twice on the 2^nd^ and 4^th^ week after PD induction(IV)) and L-dopa/carbidopa (10/30 mg/5 ml/kg, IP) (Raha daroo-Iran) for a period of four months after PD induction (18).

5) PD+D group, MFB-lesioned rats, received L-dopa/carbidopa (10/30 mg/5 ml/kg, IP) for a period of four months after PD induction. 

**Figure 1.**
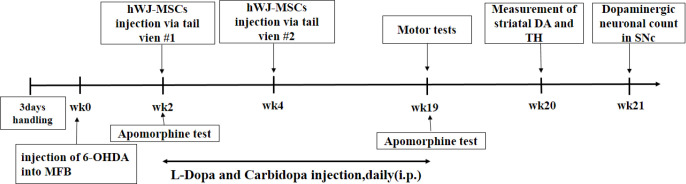
The design of research schedule and intervals to measure various parameters

After behavioral tests, each main group was divided into two subgroups (n=5) for biochemical and histological experiments, respectively. The treatment schedule and the intervals for estimation of various parameters have been presented in Figure 1. 

***Culturing of hWJ-MSCs***

**Figure 2:**
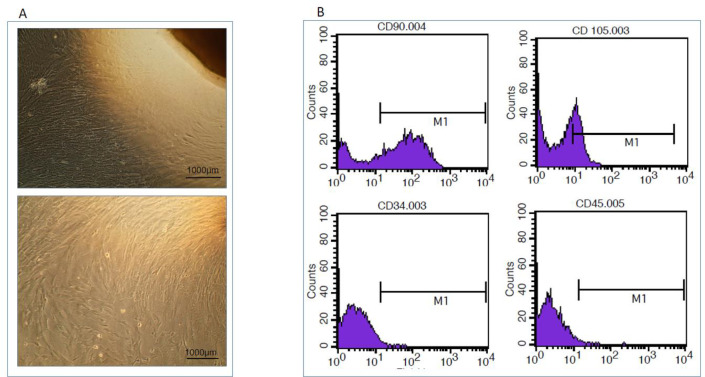
A: Undifferentiated hWJ-MSCs after three passages of culture. B: Analysis by flow cytometry: MSCs are positive for the expression of CD90, CD105, but negative for the expression of CD34, CD45

After obtaining the ethical approval code (IR.AJUMS.REC.1396.597) from the Animal Ethics Committee of AJUMS, the MSCs were collected from the Wharton’s jelly (WJ) of umbilical cords with cutting the inner matrix of WJ into 3-5 mm long pieces. The explants were cultured in the complete culture medium (CCM) containing Dulbecco’s Modified Eagle´s Medium (DMEM) (low glucose) and 2 mM L-glutamine, supplemented with 20% fetal bovine serum (FBS) and 100 IU penicillin/streptomycin. The cells were subcultured after they have reached a confluence of 80 to 90% (in about 7-10 days) (19) (Figure 2A).

***Flow cytometry***

The cells with anti-human antibodies against CD105, CD90, CD34, and CD45 were incubated for 30 min at 4^ᵒ^C. All antibodies were bought from eBioscience (San Diego, CA). Negative and isotype controls were performed. After cell staining, for each sample, 10,000 events were counted and data were analyzed using FlowJo version 8.8.7 software (Treestar, OR) (Figure 2B).

***hWJ-MSCs labeling***

In essence, cell tracker 1,1’-dioctadecyl-3,3,3’3’-tetramethylindocarbocyanine perchlorate (DiI) (c-7000) with fluorescent dye (red), which can be detected hWJ-MSCs through attaching to phospholipid membrane cells, and the labeling could last up to four months (20). The suspension was incubated at 37°C for 5 min, then at 4°C for 15 min with occasional mixing (21). hWJ-MSCs labeled were washed 3 times with DPBS before injection (1, 22).

As can be seen in the picture, a large number of labeled spindle hWJ-MSCs are seen in red, and these cells have been able to migrate from the tail vein to the lesion site in the brain.

**Figure 3:**
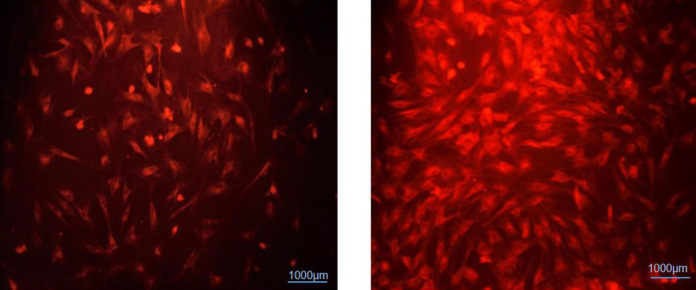
The grafted hWJ-MSCs labeled were observed in red under the fluorescence microscope (4 months after the last injection of hWJ-MSCs). 10x magnification was used for all the observations

The hWJ-MSCs labeled were conducted twice at an interval of 2 weeks into the tail vein. Rats also were received L-dopa and carbidopa (10/30 mg/kg/day, IP) simultaneously for 4 months. After 4 months all the below-mentioned behavioral tests and biochemical analysis were performed and brain sections (5 μ thick) were dissected with a cryosurgical device (SLEE, Germany) and were observed in fluorescent dye (red), which meant the migration of injected cells from the tail site to the brain (1, 3) (Figure 3). 

***Parkinson’s disease rat model***

Briefly, rats were anesthetized with a combination of ketamine/xylazine (90/10 mg/kg, IP). 6-OHDA (Sigma, USA) was prepared freshly with a concentration of 16 μg/2 μl normal saline containing 0.01% ascorbic acid (23). The neurotoxin 6-OHDA was injected into the right MFB according to the stereotaxic atlas for brain surgery with coordinates of AP: -2.2 mm to bregma, ML: + 4.7 mm to midline suture, and DV: -8.5 mm from skull surface (4, 5). 

***Apomorphine induced rotation behavior***

All the lesioned rats were tested two weeks after MFB lesion (before treatment) and 18 weeks after lesioning. Contralateral rotations of each animal were recorded after subcutaneous injection of apomorphine (0.5 mg/kg in normal saline containing 0.01% ascorbic acid) to confirm the DA depletion in the nigrostriatal system (24). The results were expressed in rotations/30 min (25).

***Muscle stiffness evaluation***

*Morprogo test*

Rats were placed on a table and their movements were scored as follows: 0, if the rat was motionless and if it was too difficult for it to move, score was 0.5. In the next step, its right and left forelimbs were respectively placed on a wooden platform (with 3 cm height). If the rat was not able to withdraw its forelimbs within 10 seconds, a score of 0.5 was given for each limb. Similar tests were conducted for the right and left forelimbs on a wooden platform of 9 cm in height. If the rat was not able to withdraw its limbs within 10 seconds, a score of 1 was given for each limb. The expected total score to fully induced the PD was 3.5, and it was less than 3.5 for a less severe disease, while the score for a healthy animal was zero (24, 26).

*Bar test*

In the bar test, the forelimbs were placed on a horizontal cylindrical metal bar (1.25 cm in diameter and 10 cm in height) and the time during which both forelimbs remained on the bar was determined in a maximum period of 10 sec. The bar test was repeated twice three and six min later, and the mean of these trials was used for analysis (27).

*Motor coordination test*

To assess motor performance and coordination, the rotarod apparatus (M.T 6800, Borj Sanat Co., Tehran, Iran) was used in all the groups. The apparatus automatically recorded the time that each rat resisted on the rotating rod. The rats were placed on a rod (at 5 rotations per min (rpm) for 3 min) to familiarize them with the instrument and the next day, the animals were placed on the rod, and speed was gradually increased to 40 rpm in 3-min intervals. The test session consisted of 3 trials in one day with 45 min intervals (cut off=300 s). Data were presented as the mean latency for bar descending (28). 

*Stride length assessment*

This device consisted of a dark wooden box with a sliding door (17×20×10 cm), a narrow tunnel (45×10×5.4 cm), and the end of the tunnel was open. The boundary between the box and the tunnel was also separated by a guillotine door. The tunnel was covered with a white paper strip and the forepaws of rats were painted with green or red ink and they were guided to walk inside the tunnel toward the dark box. The guillotine door was closed immediately after the rats entered the dark box to prevent them from returning and walking on the paper again inside the tunnel floor. Then the paper tape was removed from the tunnel to have the footprints dried and the stride length was measured as the mean of 4 consecutive footprints in each group (25).

*Cylinder test*

The rats were located in a cylindrical glass casing of 21 cm in diameter and 31 cm in height. Then, the number of times the forelimbs raised and touched the walls of the chamber was recorded for 3 min. Scoring of the test was carried out according to the following formula (28):

(Total number of contacts with the forelimbs)/(number of contacts with the forelimb of the lesion side) - (number of contacts with the forelimb of the opposite side of the lesion) × 100

*Open-field test (OFT)*

This is a general test to assess motor activity, excitability, emotionality, and exploratory behaviors in rodents (29). It consists of a square black metal box (30) with a floor that is divided by white lines into 16 equal squares 4×4 cm (31, 32). The OFT apparatus was wiped before putting a new rat in it in order to avoid any possible effect on the next rat’s behavior due to the odor that remained from the previous one (33). The behavior of each rat in the OFT was continuously recorded (video camera SONY HXR-NX100) for a period of 5 min as the observation period, and certain coded symbols were used for the following parameters: 

Ambulation frequency: The number of squares crossed by the animal (31, 34) that was scored by the total count during a 5-min period. 

Rearing frequency: The number of times the animal stood stretched on its hind limbs with and without the forelimbs support (30, 34) which was scored during a 5-min observation period.

*Measurement of brain DA and TH*

Rats with sodium pentobarbital (SIGMA-Germany) (90 mg/kg, IP) were deeply anesthetized, then hippocampi tissues were quickly removed on the ice and frozen at -80°C. In the next step, the striatum tissues were homogenized and were centrifuged (10,000 rpm, 20 min). Enzyme-linked immunosorbent assay (ELISA) kits for DA (Cat. No. ZB-ZB-10219C-R9648, Germany) and TH (Cat. No. ZB-11316C-R9648, Germany) were purchased from ZellBio GmbH (Germany). Results are reported as Pico gram of TH per milliliter (pg/ml) and nanogram of DA per liter (ng/l).

*Histological study*

The brains of rats were perfused transcardially with a neutral-buffered formalin fixative solution (NBF 10%, pH=7.4). Then, the brain tissue sections of 5 μm thick were prepared and the Nissl bodies were stained with 1% Cresyl violet for assessment of the extent of the histological lesion in the nigrostriatal pathway (35). Cell numbers were counted under a high power (×200) magnification by a light microscope. Neurons were counted only when their nuclei were clearly visualized within one focal plane. The number of neurons in SNc was expressed as the total counts obtained from the representative sections (36).

***Statistical analysis***

Data were analyzed using GraphPad Prism software version 6. The results were presented as mean±SEM and the data normality was checked using Kolmogorov–Smirnov test. The data of Morprogo test were analyzed using by Kruskal-Wallis test followed by Tukey’s *post hoc* test. Other data were analyzed by one-way ANOVA followed by Tukey’s *post hoc* test and a *P*-value less than 0.05 was considered statistically significant.

## Results

***hWJ-MSCs improved motor activity in PD rats***

*Apomorphine induced rotation test*

**Figure 4:**
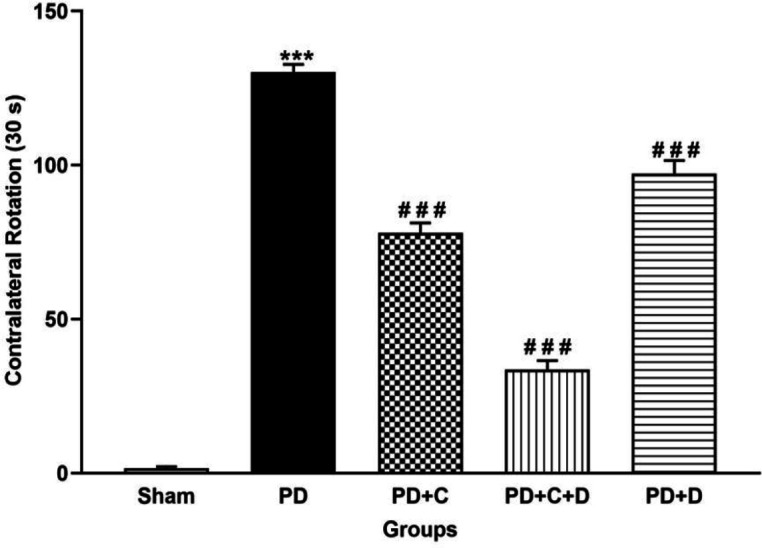
The effects of hWJ-MSCs and L-Dopa-Carbidopa on apomorphine-induced rotational test in 18 weeks after PD induction. Values are presented as mean±SEM (n=10 male Wistar rats). ***P< 0.001 versus sham group, ###*P*<0.001 versus PD group

As shown in Figure 4, 18 weeks after PD induction the number of apomorphine-induced contralateral rotations increased significantly in the PD group as compared to the sham group (F_4, 95_=298, *P*<0.001), while it was decreased significantly in treated groups (PD+C, PD+C+D, and PD+D groups) versus PD (*P*<0.001).

*Bar and muscle stiffness tests*

**Figure 5:**
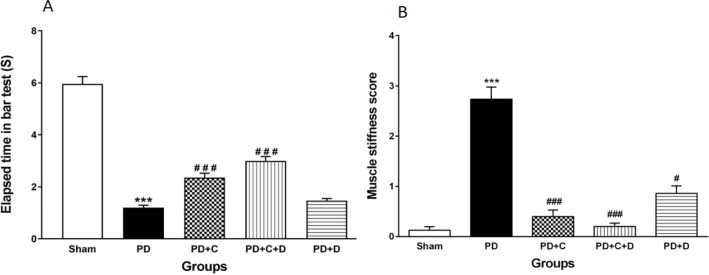
Administration of hWJ-MSCs with L-Dopa-Carbidopa on motor activity of different tested groups in the bar test. Elapsed time (s) was increased significantly in PD group vs. sham group (*P*<0.001), while it was reversed significantly in PD+C and PD+C+D groups (*P*<0.001). Furthermore, no perceptible difference was observed between the PD and PD+D groups (A). The Morpurgo’s test that was used to evaluate muscle stiffness after PD induction (n=10 male Wistar rats) (B). Muscle stiffness score was significantly increased in PD in comparison with the sham group (*P*<0.001), while it was decreased significantly in PD+C, PD+C+D groups (*P*<0.001), and PD+D group (*P*<0.05) vs. the PD group. Data was analyzed by the nonparametric rank sum approach of Kruskal-Wallis test. Symbols***P<0.001 versus sham group and ###*P*<0.001 and #*P*<0.05 vs. PD group

As shown in Figure 5B, the latency in bar test significantly increased in the PD group as compared to the sham group (F_4, 95_=121, *P*<0.001), while it significantly decreased in PD+C and PD+C+D groups (*P*<0.001) versus the PD group. On the other hand, no perceptible difference was observed between the PD and PD+D groups. 

As shown in Figure 5B, muscle stiffness score as an index of catalepsy in Morprogo test was significantly increased in PD rats in comparison with the sham group (*P*<0.001), while it was significantly decreased in PD+C and PD+C+ D groups compared with PD (*P*<0.001), but in PD+D it was similar to PD (*P*<0.05).

*Motor coordination test*

**Figure 6:**
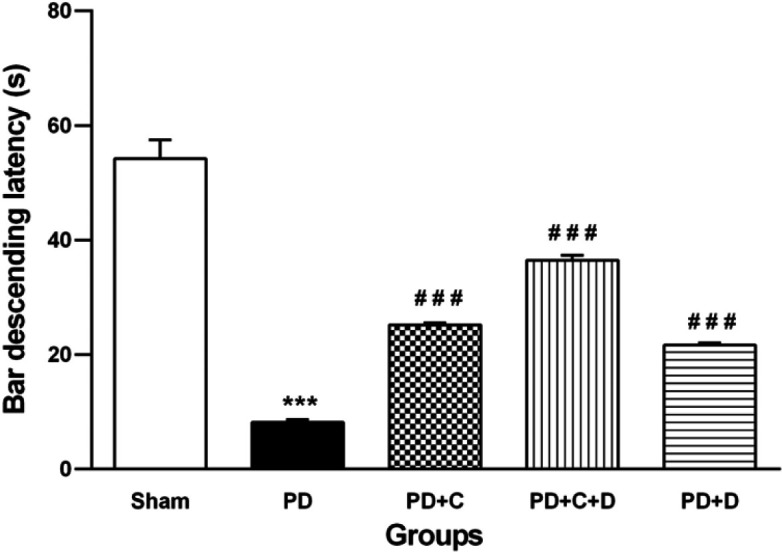
Effects of hWJ-MSCs and L-Dopa-Carbidopa on bar descent latency of the rotarod. (mean ± SEM; One-way ANOVA and Tukey’s test (n=10 male Wistar rats). Bar descending latency was reduced significantly in PD group vs. sham group (*P*<0.001) while it was significantly increased in PD+C, PD+C+D and PD+D groups (*P*<0.001) vs. PD group. Symbols *** and ### vs. sham and PD groups, respectively

Motor coordination in the PD group showed a significant decrease compared to the sham group (F _4,95_=123, *P*<0.001), and treatment with hWJ-MSCs and L-dopa-carbidopa could increase the motor coordination significantly in PD+C, PD+D, and PD+C+D groups versus the PD group (*P*<0.001) (Figure 6).

*Stride length assessment*

**Figure 7:**
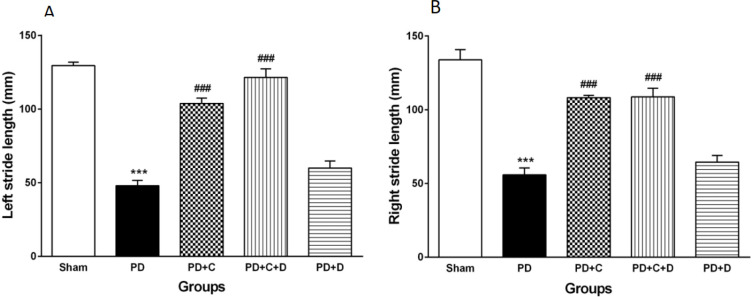
Administration of hWJ-MSCs with L-Dopa-Carbidopa on stride-length. (mean ± SEM; One-way ANOVA and Tukey’s test (n=10 male Wistar rats). In the PD group, the left stride length (A) and the right stride length (B) of forepaws were significantly decreased in the sham group (*P*<0.001), while they increased in the PD+C and PD+C+D groups (*P*<0.001) vs. PD group. Furthermore, no perceptible difference was observed between the PD and PD+D groups. Symbols *** and ### vs. sham and PD groups, respectively

The stride length of forepaws (left and right) of experimental groups has been shown in Figures 7A, B. It was significantly decreased in PD rats as compared to the sham group (F_4, 95_=76.6, *P*<0.001), while it was increased significantly in both PD+C and PD+C+D groups versus the PD group (*P*<0.001). Furthermore, no perceptible difference was observed between PD+D and PD groups.

*Cylinder test*

**Figure 8:**
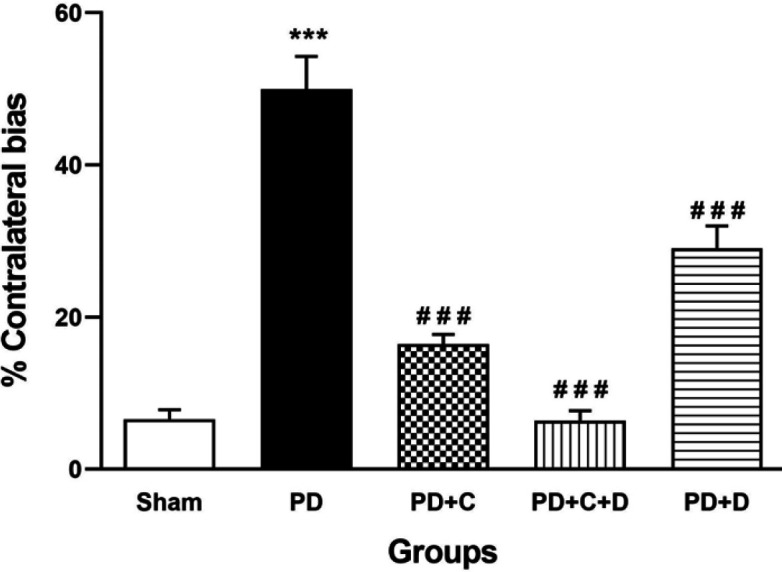
Administration of hWJ-MSCs and L-Dopa-Carbidopa on the scores of cylinder test. mean ± SEM; One-way ANOVA and Tukey’s test (n=10 male Wistar rats). The score of cylinder test was increased significantly in PD group vs. sham group (*P*<0.001) while it was reduced significantly in PD+C, PD+C+D, and PD+D groups (p<0.001) vs. PD group. ***P<0.001 vs. sham group and ###*P*<0.001 vs. PD

The scores of the cylinder test were significantly increased in PD rats versus the sham group (F_4, 95_=52.7, *P*<0.001). On the other hand, they had a significant reduction in PD+C, PD+D, and PD+C+D groups versus the PD group (*P*<0.001, Figure 8). 

*Locomotion test*

**Figure 9:**
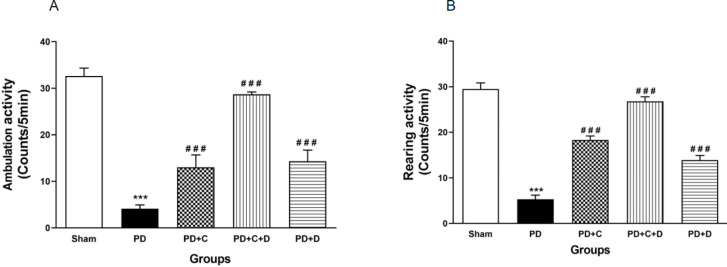
Administration of hWJ-MSCs and L-Dopa-Carbidopa on locomotor activity of different tested groups in open field test. (mean ± SEM; One-way ANOVA and Tukey’s test (n=10 male Wistar rats). The ambulation or line crossing (A) was reduced significantly in PD group versus sham group (*P*<0.001), while it was significantly increased in PD+C, PD+D and PD+C+D groups (*P*<0.001) vs. PD group. The rearing as an exploratory behavior (B) had significantly reduction in PD group vs. sham group (*P*<0.001), while it was significantly increased in PD+C, PD+D and PD+C+D groups (*P*<0.001) vs. PD group. ***P<0.001 vs. sham group and ###*P*<0.001 vs. PD

The ambulation and rearing frequencies in the open field test have been shown in Figure 9A, B. They were significantly decreased in PD rats versus the sham group (F_4, 45_=133.7, *P*<0.001) (F_4, 45_=90.38, *P*<0.001) respectively, in contrast to the sham group. These behaviors in PD+C, PD+D, and PD+C+D groups were increased significantly versus the PD group (*P*<0.001).

*Brain DA and TH level*

**Figure 10:**
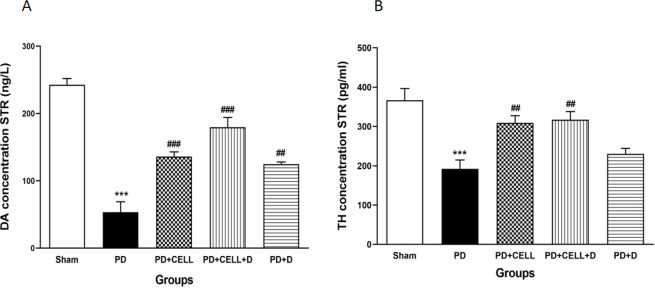
Administration of hWJ-MSCs and L-Dopa-Carbidopa on dopamine level (A) and tyrosine hydroxylase (B) in the striatum. (mean ± SEM; One-way ANOVA and Tukey’s test (n=10 male Wistar rats). The levels of TH and DA of the PD group were considerably reduced in comparison to the sham group (*P*<0.001). The levels of DA increased significantly in the PD+C, PD+C+D, and PD+D groups (*P*<0.001, *P*<0.01) compared to the PD group. Contents of TH were increased significantly in the PD+C and PD+C+D groups (*P*<0.01). However, no perceptible difference was observed in TH content between the PD and PD+D groups. ***P<0.001 vs. sham group, ###*P*<0.001, ##*P*<0.01 vs. PD group

As shown in Figure 10A, B, the levels of TH (pg/ml) and DA (ng/l) in the PD rats were considerably decreased (F_4, 20_=11.48, *P*<0.001) (F_4, 20_=40.26, *P*<0.001) respectively versus the sham group (*P*<0.001). Moreover, TH contents were increased significantly in the PD+C and PD+C+D groups (*P*<0.01). Although, no perceptible difference was observed between the PD and PD+D groups. In addition, DA level was increased significantly in PD+C+D, PD+C (*P*<0.001), and PD+D groups (*P*<0.01) compared to the PD rats.

*Neuronal count in SNc*

**Figure 11:**
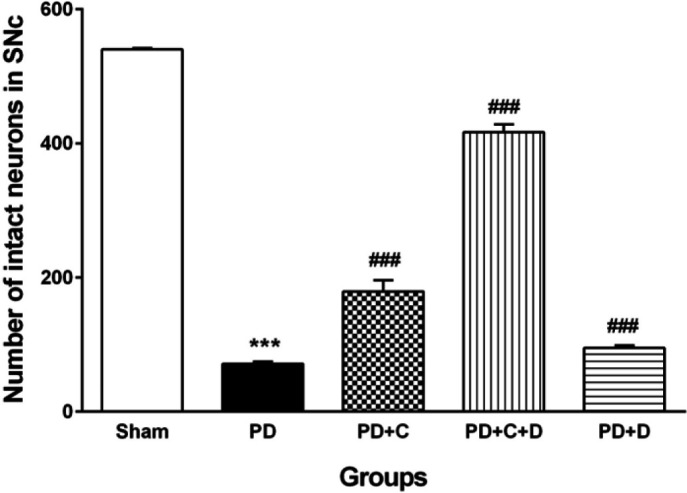
Effects of hWJ-MSCs and L-Dopa-Carbidopa on the number of dopaminergic cells in the SNc. (mean ± SEM; One-way ANOVA and Tukey’s test (n=10 male Wistar rats). By comparing, the number of dopaminergic cells in a square millimeter area of the SNc, a significant reduction was found in the PD group in comparison with the sham group (*P*<0.001). However, the number was increased in the PD+C, PD+C+D, and PD+ D groups vs. the PD group (*P*<0.001). ***P<0.001 vs. sham group, ###*P*<0.001 vs. PD group

**Figure 12:**
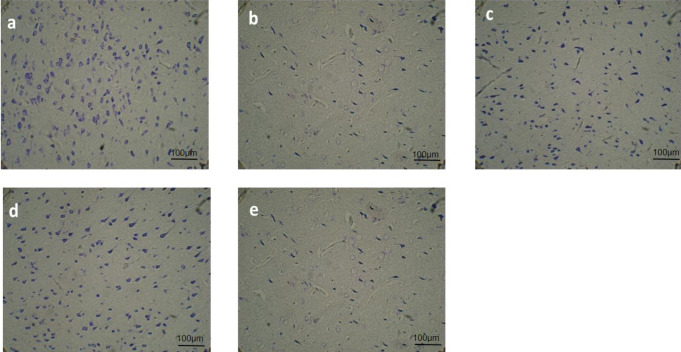
The histological study by Nissl staining with 0.1% crystal violet in all the tested groups. Photomicrographs of coronal sections (5 micrometers) of the SNc (n=5 male Wistar rats). a: sham; b: PD; c: PD+C; d: PD+C+D; and e: PD+D. The reduction of dopaminergic cells is visible in the PD group in comparison with the sham group (*P*<0.001). 6-OHDA-induction further decreased the nigrostriatal neurons in the PD group and administration of hWJ-MSCs and L-Dopa-Carbidopa prevented from the dopaminergic cells reduction so the slide of the PD+C+ D group (d) has a perspective close to that from the sham group (*P*=0.0744)

Histological evaluation showed that the counted neurons in the different tested groups were varied significantly (F_4, 45_=489, *P*<0.001). The number of neurons in the SNc of the PD animals was considerably reduced as compared to the sham group (*P*<0.001). The SNc in PD+C, PD+D, and PD+C+D groups appeared to contain substantially more dopaminergic neurons as compared to the PD group (*P*<0.001) (Figure 11). In addition, Figure 12 indicated the density of the dopaminergic neurons of the SNc. 

## Discussion

Our findings showed that hWJ-MSCs could restore motor impairments and increased the brain DA and TH levels as well as the number of DA neurons in SNc, which lead to improving the motor disturbances. However, the best results were found in the groups that received both of hWJ-MSCs and L-dopa-carbidopa; therefore, It can be concluded that transplantation of hWJ-MSC with levodopa can be a good strategy to improve motor behaviors in Parkinson’s disease.

PD afflicts primarily the dopaminergic neurons, which have their cell bodies located in SNc. Many current treatments of PD can only address the symptoms but not the underlying neurodegenerative mechanisms of PD (37). 

Chronic treatment of PD with levodopa is often characterized by the progress of various types of movement response oscillations during the day as well as drug-induced dyskinesia. Such treatment-related motor complications eventually develop in most of the patients. In severe cases, treatment-induced dyskinesias may completely eliminate the therapeutic benefit originally gained from the drug (38). As shown in the above results, the decreased therapeutic effects of levodopa in group PD+D were also seen compared to the other two treatment groups.

The aim of cell therapy for PD is the replacement of dopaminergic neurons in the SN with stem cells or the prevention of these neurons from further reduction (39). The latest experiments have shown that MSCs by autophagy modulation (40) and dopaminergic carotid body grafts have neuroprotective effects and could prevent SN dopaminergic neurodegeneration in animal models of PD (41). hMSCs in WJ of the umbilical cord can be easily obtained, and have a rapid growth rate in culture and long-term survival, and can be used in PD (3). 

In the current study, the possible effect of WJ-MSCs for PD treatment was confirmed using the intravenous transplantation of hWJ-MSCs in the 6-OHDA-induced PD rat model. Injected hWJ-MSCs improved motor disturbances in cylinder, bar, rotarod, Morpurgo’s, open field, rotation, and forepaws stride-length tests in the PD rat model. Gait disorders are commonly observed in patients with PD and occur due to the reduction of dopaminergic neurons in the SN (42). In addition, according to several studies, it is cleared that the role of mesocorticolimbic network and associated frontostriatal projection areas in the motivational aspects of behavior and injury in this network may contribute to a reduced drive to explore (43-45). Administration of WJ-MSCs (IV) could be a more appropriate method for damage recovery than direct implantation into the brain (46). Overall, the oxidative stress caused by 6-OHDA leads to dopaminergic neuronal cell death. However, the 6-OHDA-induced rat model of PD that is similar to human PD in its sever-stage symptoms, may be suitable for the studies of cell therapy through replacing cells after neuronal loss which occurs due to aging (47-50). Some studies have suggested that hWJ-MSCs have the potential to treat PD (3). Moreover, adipose-derived MSCs increase sub-ventricular neurogenesis in the PD rats of the 6-OHDA model (16). 

Measuring the concentration of DA and TH showed increased striatal levels in the treated PD groups. The rising of TH in the striatum can increase the level of DA in the brain and improve apomorphine-induced rotation, which is beneficial in the treatment of PD (51). Another plausible hypothesis is that the injected cells produced glial cell line-derived neurotrophic factor (GDNF), which then induced surviving striatal axons and terminals to grow into the lesioned region, thereby increasing the DA level in the striatum (52-55). Huang* et al*. in 2012 demonstrated that transplantation of TH and neurturin gene-modified bone marrow-derived MSCs increase DA synthesis and significantly improve the motor activity of PD rats (56).

In the WJ-MSCs-transplanted PD rat brains, the cell population of Nissl stained was increased in the SNc. These histological findings confirmed that WJ-MSCs had led to the relevant behavioral improvements and increased the total number of neurons in the SNc in the treated groups, suggesting that the number of transplanted dopaminergic neurons is an important factor in the treatment of PD (3). 

Consistently, these findings revealed that hWJ-MSCs have an improving motor function by proliferation and differentiation to nerve cells in PD rats. Although, further studies are essential to explain the neuroprotective mechanisms of hWJ-MSCs.

Sham: The group received vehicle of 6-OHDA; PD: The group received 6-OHDA; PD+C: The group received hWJ-MSCs; PD+C+D: The group received hWJ-MSCs and also L-Dopa/Carbidopa; PD+D: The group received L-Dopa/Carbidopa

Sham: The group received vehicle of 6-OHDA; PD: The group received 6-OHDA; PD+C: The group received hWJ-MSCs; PD+C+D: The group received hWJ-MSCs and also L-Dopa/Carbidopa; PD+D: The group received L-Dopa/Carbidopa

Sham: The group received vehicle of 6-OHDA; PD: The group received 6-OHDA; PD+C: The group received hWJ-MSCs; PD+C+D: The group received hWJ-MSCs and also L-Dopa/Carbidopa; PD+D: The group received L-Dopa/Carbidopa

Sham: The group received vehicle of 6-OHDA; PD: The group received 6-OHDA; PD+C: The group received hWJ-MSCs; PD+C+D: The group received hWJ-MSCs and also L-Dopa/Carbidopa; PD+D: The group received L-Dopa/Carbidopa

Sham: The group received vehicle of 6-OHDA; PD: The group received 6-OHDA; PD+C: The group received hWJ-MSCs; PD+C+D: The group received hWJ-MSCs and also L-Dopa/Carbidopa; PD+D: The group received L-Dopa/Carbidopa

Sham: The group received vehicle of 6-OHDA; PD: The group received 6-OHDA; PD+C: The group received hWJ-MSCs; PD+C+D: The group received hWJ-MSCs and also L-Dopa/Carbidopa; PD+D: The group received L-Dopa/Carbidopa

Sham: The group received vehicle of 6-OHDA; PD: The group received 6-OHDA; PD+C: The group received hWJ-MSCs; PD+C+D: The group received hWJ-MSCs and also L-Dopa/Carbidopa; PD+D: The group received L-Dopa/Carbidopa

Sham: The group received vehicle of 6-OHDA; PD: The group received 6-OHDA; PD+C: The group received hWJ-MSCs; PD+C+D: The group received hWJ-MSCs and also L-Dopa/Carbidopa; PD+D: The group received L-Dopa/Carbidopa

Sham: The group received vehicle of 6-OHDA; PD: The group received 6-OHDA; PD+C: The group received hWJ-MSCs; PD+C+D: The group received hWJ-MSCs and also L-Dopa/Carbidopa; PD+D: The group received L-Dopa/Carbidopa

## Conclusion

The present study shows that the hWJ-MSCs alone and/or with a combination of common medication such as L-dopa/carbidopa could be one of the ways for the treatment of PD. However, more experiments are required in order to find a better understanding of the mechanism(s) involved in pathogenesis and treatment strategies of Parkinson’s patients with hWJ-MSCs. 

## Authors’ Contributions

MJ analyzed the data and contributed to writing the manuscript. AS was responsible for monitoring and approving behavioral tests in different experimental groups. YF was responsible for confirming the 6-OHDA-induced PD. SZ was responsible for hWJ-MSCs labeling, migration of injected cells, and revising the article. EM was responsible for histological evaluations. MD performed and analyzed the examination of the biochemical factors. GHS designed, guided, and supervised the project and monitored hWJ-MSCs isolation and transplantation.

## Conflicts of Interest

The authors declare that they have no conflict of interest.
